# Oral Contraceptives and Multiple Sclerosis/Clinically Isolated Syndrome Susceptibility

**DOI:** 10.1371/journal.pone.0149094

**Published:** 2016-03-07

**Authors:** Kerstin Hellwig, Lie H. Chen, Frank Z. Stancyzk, Annette M. Langer-Gould

**Affiliations:** 1 St. Josef University Hospital, Bochum, Neuroimmunological Ward and Outpatient Clinic, Bochum, Germany; 2 Kaiser Permanente Southern California, Department of Research & Evaluation, Pasadena, California, United States of America; 3 University of Southern California, Keck School of Medicine, Los Angeles, California, United States of America; 4 Kaiser Permanente, Southern California Los Angeles Medical Center, Neurology Department, Los Angeles, California, United States of America; Strasbourg university hospital, FRANCE

## Abstract

**Background:**

The incidence of multiple sclerosis (MS) is rising in women.

**Objective:**

To determine whether the use of combined oral contraceptives (COCs) are associated with MS risk and whether this varies by progestin content.

**Methods:**

We conducted a nested case-control study of females ages 14–48 years with incident MS or clinically isolated syndrome (CIS) 2008–2011 from the membership of Kaiser Permanente Southern California. Controls were matched on age, race/ethnicity and membership characteristics. COC use up to ten years prior to symptom onset was obtained from the complete electronic health record.

**Results:**

We identified 400 women with incident MS/CIS and 3904 matched controls. Forty- percent of cases and 32% of controls had used COCs prior to symptom onset. The use of COCs was associated with a slightly increased risk of MS/CIS (adjusted OR = 1.52, 95%CI = 1.21–1.91; p<0.001). This risk did not vary by duration of COC use. The association varied by progestin content being more pronounced for levenorgestrol (adjusted OR = 1.75, 95%CI = 1.29–2.37; p<0.001) than norethindrone (adjusted OR = 1.57, 95%CI = 1.16–2.12; p = 0.003) and absent for the newest progestin, drospirenone (p = 0.95).

**Conclusions:**

Our findings should be interpreted cautiously. While the use of some combination oral contraceptives may contribute to the rising incidence of MS in women, an unmeasured confounder associated with the modern woman’s lifestyle is a more likely explanation for this weak association.

## Introduction

Whether oral contraceptives (OC) influence the risk of multiple sclerosis (MS) is unclear. The most popular hypothesis is that OCs should protect against MS because pregnancy (a high estrogen and progesterone state) ameliorates disease activity in humans as does exogenous administration of high-dose estrogens in the animal model of MS. However, the recently reported rising incidence of MS in women but not men[1] have led others to hypothesize that OC use could be partially responsible[1], along with other factors that reflect the modern woman’s lifestyle.

The most commonly used forms of hormonal contraception today are combined oral contraceptives (COCs) containing low doses of estrogen and one of a variety of progestins. The effects of these low dose hormones either individually or in combination on animal models of MS have not been carefully studied. Progestins are particularly interesting because they vary in their affinity for androgen, progesterone and glucocorticoid receptors and could therefore have theoretically diverse effects on MS susceptibility.

The sparse number of previous studies of COC use and MS risk hypothesized a protective effect and concluded that COCs had either no effect[2–4] or a protective effect(5). However, two of these studies reported a statistically non-significant trend toward a slight increase[2, 3] in MS risk. These studies had several limitations included long enrollment period[2], small samples[3–5] and/or non-representative samples[2–4]. None examined whether the risk varies by progestin content.

The purpose of this study was to determine whether use of contemporary COCs could be influencing the risk of MS and whether this risk varies by progestin content.

## Methods

### Study Population

This nested case-control study was based on a retrospective cohort of women of childbearing age (14–48 years) who were members of Kaiser Permanente Southern California (KPSC) health plan between Jan 1, 2008 and Dec 31, 2011.

### Setting

The institutional review board (IRB) at Kaiser Permanente Southern California (KPSC) approved this study. Informed consent by the IRB was waived as this was a chart review study only without direct patient contact. Analyses were performed on a de-identified limited dataset. KPSC is a large prepaid health maintenance organization with over 3.5 million members. It provides comprehensive health care coverage to ~20% of the population in the geographic area it serves[6]. The KPSC membership is representative of the general population in Southern California with respect to ethnicity, age, gender and socioeconomic status with the exception of an under-representation of the lowest and highest ends of the socioeconomic spectrum[6]. The cost of specialist consultations, hospitalizations, MRI scans, other diagnostic tests and medications are almost fully covered with minimal co-pays. This study utilized data from the electronic clinical databases including the pharmacological, clinical and administrative records.

### Case Identification

We used the same methods as described in detail elsewhere[7, 8] to identify individuals with newly diagnosed MS or its potential precursor, clinically isolated syndrome (CIS). Briefly, we searched electronic medical records for first mention of ICD-9 diagnostic codes for MS and other acquired CNS demyelinating diseases January 1, 2008–December 31, 2011 including all inpatient and outpatient encounters since enrollment into the health plan (N = 3556). Diagnoses were confirmed, symptom onset date and additional clinical details were extracted through full medical records abstraction including all inpatient and outpatient records, MRI scans, and diagnostic test results by an MS specialist (ALG) according to revised McDonald criteria for MS[9] and the proposed consensus definitions for idiopathic transverse myelitis (TM)[10, 11]. All patients with optic neuritis (ON) had been evaluated by ophthalmologists who confirmed the diagnosis. Men and females less than 14 or older than 48 years old were excluded. Cases with symptom onset prior to January 1, 2000, missing/imprecise symptom onset date or those without 12 months of continuous KPSC membership prior to symptom onset were excluded.

### Control Selection

For each incident case, a maximum of 10 control subjects from the KPSC population were matched to the case on date of birth (within 1 year), sex, race/ethnicity and membership characteristics including membership duration (within 1 year) at the time of the case’s symptom onset date to avoid immortal time bias. The matched controls were assigned the same index date as their matched case (symptom onset date) and were also required to have 12-month continuous membership prior to the index date for study inclusion. With this algorithm 99% of cases had 10 matched controls.

### Hormonal Contraceptive Use

Pharmacy records within 10 years of index date were obtained from the complete electronic health records (EHR). The search included product codes indicating prescriptions of contraceptive use in pharmacy database, and current procedural terminology (CPT) procedure codes relating to contraceptive use in clinical database. The pharmacy database includes the inpatient and outpatient prescription medication dispensed at KP hospitals, medical centers and medical offices. Ninety seven percent of members have a drug benefit with minimal co-pays and fill their prescriptions at Kaiser Pharmacies located at each facility.

Combination oral contraceptive (COC) use was defined in the following forms: (1) ever vs. never use, (2) current, former, and never use (3) duration or cumulative dose. Ever use was defined as two and more consecutive COC prescriptions of any type covering at least 56 days to avoid misclassifying women who did not continue COCs for at least 1 cycle. Current users were defined as ever users who had at least one prescription period prior to and within 1 month of the index date. Former users were defined as ever users who had no dispense periods prior to and within 1 month of the index date. Duration of use was calculated by dispense date, and supply days. Duration of use was modeled as categorical variables based on the quintiles of duration use in controls up to ten years prior to index date.

**Covariates** extracted from KPSC EHR and membership files included the most recent body mass index (BMI) and smoking status recorded prior to the index date; and births and miscarriages/abortions in the 10 years before the index date. 88 controls were excluded from analyses due to missing BMI information.

### Statistical analyses

Conditional logistic regression was used to estimate the matched odds ratio (OR) and its corresponding 95% confidence interval (CI) for the association between MS/CIS and HC and COC use. The models were adjusted for BMI (under or normal weight ≤ 25.0 kg/m^2^, over-weight25-29.99 kg/m^2^, class I-III obesity class I 30–34.99 kg/m^2^; class II 35–39.99 kg/m^2^; class III ≥40.0 kg/m^2^), smoking (ever/never), live births (0, 1 or more) and abortions/miscarriages (yes/no). Analyses of COC use were stratified by progestin family as follows norethindrone (norethindrone, norethindrone acetate, ethynodiol diacetate); levonorgestrel (levonorgestrel, norgestimate, norgestrel, desogestrel) and drospirenone. Women who used multiple types of COCs with different progestin family contents were classified in two ways: the most recently or longest used COC family during the study period. Sensitivity analyses of the primary and secondary exposure (HC and COC use ever/never) restricted to 3 years prior to symptom onset or MS cases only were conducted. Post-hoc analyses restricted to MS cases only were conducted.

The means and standard deviations of normally distributed continuous variables were compared using two-sample *t* tests; Wilcoxon-Mann-Whitney test for non-normally distributed continuous variables and for binary or categorical variables, Chi-square or Fisher exact test. All analyses were conducted using SAS software v9.2 (Cary, NC).

## Results

In all, 400 women ages 14–48 years with newly diagnosed MS or CIS were included in the study after exclusion of 5 individuals (1%) because their symptom onset could not be precisely determined and 126 (23.7%) because they had less than 12 months of KPSC membership prior to symptom onset. Of these, 239 (60%) met diagnostic criteria for MS and 113 (70%) of the 161 CIS cases had abnormal brain MRI scans at the time of symptom onset indicating a high lifetime risk of MS. Most cases (n = 363, 91%) had onset of symptoms between 2007 and 2011.

The baseline demographic and clinical characteristics of cases and controls are presented in Table 1.

**Table 1:** Baseline demographic and clinical characteristics.

	Cases (n = 400)		Controls (n = 3904)		p-value
	n	%	n	%	
Age (year), mean (SD)	33.8	9.2	33.8	9.2	0.970
Race/Ethnicity, n (%)					0.986
White	157	39.3	1539	39.4	
Hispanic	130	32.5	1284	32.9	
Blacks	88	22.0	853	21.8	
Asian/PI	17	4.3	165	4.2	
Other	8	2.0	63	1.6	
Body Mass Index, n (%)					0.104*
Under/normal weight	130	32.5	1429	36.6	
Overweight	118	29.5	1115	28.6	
Obesity Class I-III	152	38.0	1360	34.8	
Smoking, n (%)					0.243
ever	115	28.8	1017	26.1	
never	285	71.3	2887	73.9	
Parity, n (%)					0.001
0	334	83.5	2940	75.3	
1	43	10.8	640	16.4	
2+	23	5.8	324	8.3	
Miscarriage (1+), n (%)	30	7.5	400	10.2	0.081
Membership duration (months)					
Ever COC users, n	160		1273		0.468
median (range)	114.5	(13.0–120.0)	111	(12.0–120.0)	
Never COC users, n	240		2719		0.026
median (range)	90.5	(13.0–120.0)	89	(12.0–120.0)	

Abbreviations COC = combined oral contraceptives; PI = Pacific Islanders

*under/normal weight vs. overweight or obese

Women with MS/CIS were more likely to be obese and have smoked but less likely to have given birth or had a miscarriage/abortion. Matching factors including age, race/ethnicity and membership duration was similar among cases and controls. Women classified as never COC users had a shorter duration of membership than ever users but this did not vary by case/control status (Table 1).

Combined oral contraceptives were the most commonly used type of hormonal contraceptive. Very few women used progestin-only oral contraceptives (13 cases and 140 controls), non-oral agents (patch 14 cases and 140 controls; vaginal ring 16 cases and 128 controls) or high dose estrogen (50μg) COCs (2 cases and 18 controls) precluding further analysis (Table 2).

**Table 2:** Characteristics of hormonal contraceptive use.

	Case (n = 400)		Control (n = 3904)		p-
	N	%	N	%	value
Any hormonal Contraceptive					
Never	224	56.0	2460	63.0	**0.006**
Ever (including current+former)	176	44.0	1444	37.0	
Current	56	14.0	441	11.3	**0.021**
Former	120	30.0	1003	25.7	
Combination OC					
Never	240	60.0	2644	67.7	**0.002**
Ever (including current+former)	160	40.0	1260	32.3	
Current	51	12.8	405	10.4	**0.007**
Former	109	27.3	855	21.9	
Combination OCs Duration (months)	**median**	**range**	**median**	**range**	
Never	2.8	(0.9–3.3)	2.8	(0.0–3.3)	
Quintile 1	5.5	(1.8–7.9)	5.5	(1.2–8.0)	
Quintile 2	10.6	(8.0–12.0)	11	(8.0–15.3)	
Quintile 3	15.7	(12.0–21.1)	20	(15.3–25.9)	
Quintile 4	30.6	(21.5–42.9)	34.4	(26.1–46.0)	
Quintile 5	69.3	(43.1–111.9)	67.9	(46.1–114.3)	

**Fig 1:**
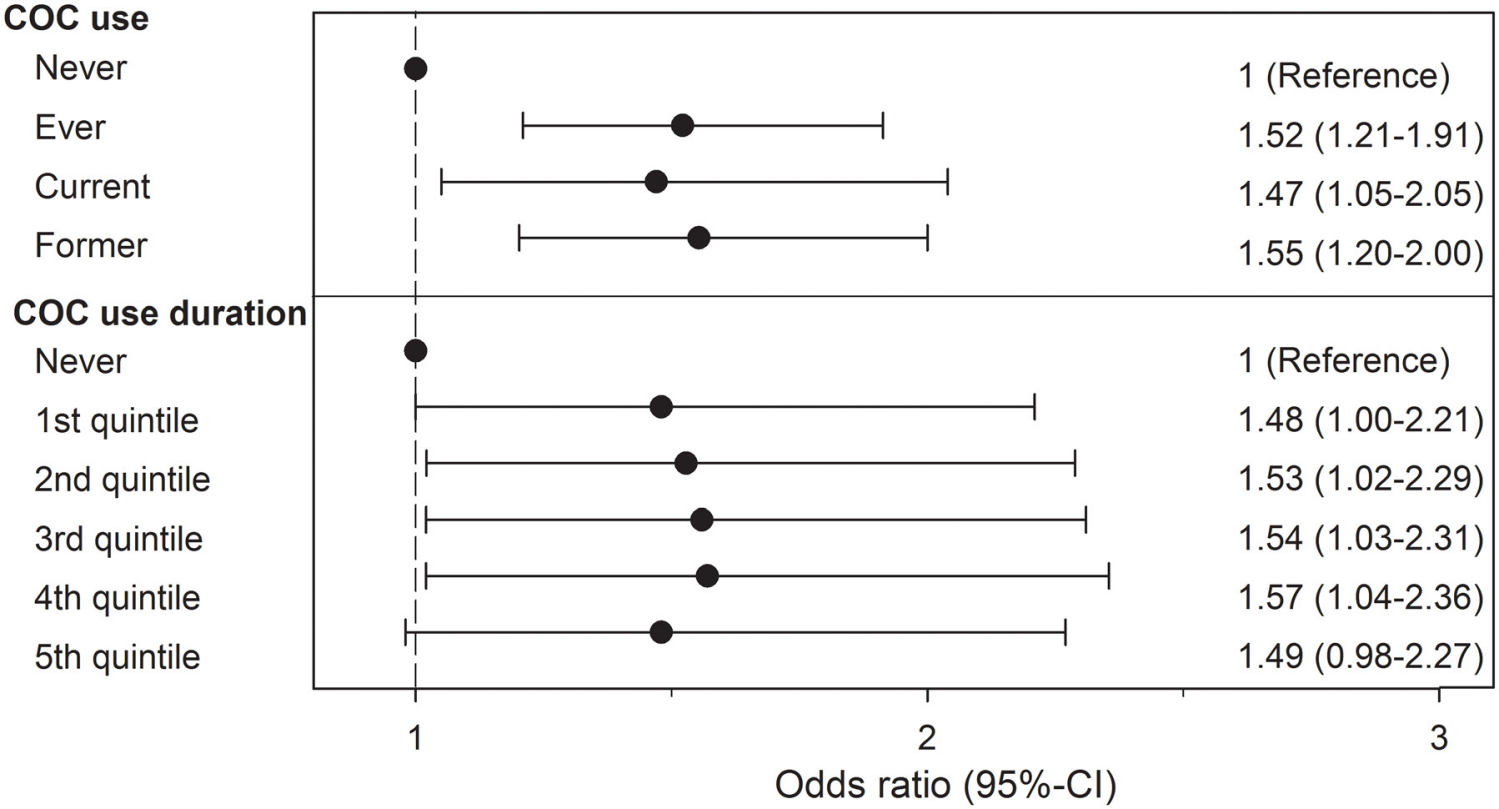
Association between combined oral contraceptive use and multiple sclerosis/clinically isolated syndrome. Ever using COCs, regardless whether current or former, was associated with a slight increased risk of MS/CIS (p<0.001; p = 0.02; and p = 0.001 respectively). Longer duration of use (1^st^shortest -5^th^longest quintiles) did not result in any change in risk. OR are adjusted for parity, miscarriage/abortions, smoking and obesity.

The use of COCs was independently associated with a slight increased risk of MS/CIS regardless of whether it was current or former use (Fig 1). Analyses restricted to those women with MS yielded similar results (adjusted OR = 1.51, 95%CI 1.12–2.03; p = 0.007, ever use; Table 3). Analyses restricted to women with at least 3 years of continuous membership prior to symptom onset (n = 305) also yielded similar results (adjusted OR = 1.35, 95%CI 1.01–1.80; p = 0.04 ever use).

**Table 3:** Association between combined oral contraceptives and MS (n = 239 cases/2322 controls).

		Crude				Adjusted^a^		
	OR	95% CI		p-value	OR	95% CI		p-value
Combined Oral Contraceptive								
Ever	1.47	1.09	1.98	0.011	1.51	1.12	2.03	0.007
Never (reference)	1.00				1.00			
Parity								
1+	0.53	0.36	0.77	0.001	0.54	0.36	0.8	0.002
None (reference)	1.00				1.00			
Miscarriage/Abortion								
1+	0.71	0.42	1.17	0.179	0.87	0.51	1.47	0.591
None (reference)	1.00				1.00			
Smoking								
Ever	1.42	1.05	1.92	0.024	1.38	1.02	1.86	0.04
Never (reference)	1.00				1.00			
Body Mass Index								
Obesity class I-III	1.02	0.73	1.41	0.921	1.04	0.75	1.45	0.821
Overweight	0.94	0.67	1.33	0.732	0.96	0.68	1.35	0.811
Normal/Underweight (reference)	1.00				1.00			

^a^. Adjusted for parity, miscarriage, smoking, obesity

The cumulative duration of use of COCs up to 10 years prior to symptom onset/index date ranged from 1 to 114 months (Table 2). There was no clear trend in MS risk with increasing duration of use when comparing increasing quintiles of duration of COC use to never users (reference category; Fig 1). Being nulliparous up to ten years prior to symptom onset and obesity were independent risk factors for MS/CIS although the latter did not reach statistical significance (Table 4). When analyses were restricted to only those women with MS, parity and smoking but not obesity were independently associated with MS (Table 3).

**Table 4:** Association between combined oral contraceptives and MS/CIS.

		Crude				Adjusted^a^		
	OR	95% CI		p-value	OR	95% CI		p-value
Combined Oral Contraceptive								
Ever	**1.48**	**1.18**	**1.85**	**<0.001**	**1.52**	**1.21**	**1.91**	**<0.001**
Never (reference)	1.00				1.00			
Parity								
1+	**0.54**	**0.40**	**0.73**	**<0.001**	**0.55**	**0.40**	**0.75**	**<0.001**
None (reference)	1.00				1.00			
Miscarriage/Abortion								
1+	0.71	0.48	1.05	0.085	0.85	0.57	1.28	0.442
None (reference)	1.00				1.00			
Smoking								
Ever	1.17	0.92	1.49	0.19	1.13	0.89	1.44	0.318
Never (reference)	1.00				1.00			
Body Mass Index								
Obesity class I-III	1.26	0.97	1.63	0.079	1.28	0.99	1.66	0.064
Overweight	1.18	0.91	1.54	0.221	1.20	0.92	1.57	0.174
Normal/Underweight (reference)	1.00				1.00			

^a^. Adjusted for parity, miscarriage, smoking, obesity

Many women (48%) used COC preparations that varied in progestin family content. The most common progestins used were norethindrone (NE) and levonorgestrol (LNG; 56.9% and 44.6% of COC users, respectively). Both NE family and LNG family-containing COCs were associated with a modest increased MS/CIS risk although this risk was more pronounced among LNG users regardless if women were classified by which type they used most recently or the longest (Table 5). Interestingly, using drospirenone-containing COCs was not associated with MS/CIS.

**Table 5:** Association between Progestin Content of COC and MS/CIS.

	Case/Control		Crude				Adjusted^a^		
	n	OR	95% CI		p-value	OR	95% CI		p-value
Family of progestins most recently used COCs^b^									
None (reference)	224/2460	1.00							
Norethindrone	69/536	**1.49**	**1.10**	**2.00**	**0.009**	**1.57**	**1.16**	**2.12**	**0.003**
Levenorgestrol	69/471	**1.71**	**1.27**	**2.31**	**<0.001**	**1.75**	**1.29**	**2.37**	**<0.001**
Drospirenone	22/253	1.01	0.64	1.62	0.955	1.02	0.64	1.62	0.950
Family of progestins longest used COCs									
None	224/2460								
Norethindrone	69/576	**1.38**	**1.03**	**1.86**	**0.034**	**1.45**	**1.08**	**1.96**	**0.015**
Levenorgestrol	70/468	**1.77**	**1.31**	**2.38**	**<0.001**	**1.82**	**1.34**	**2.46**	**<0.001**
Drospirenone	21/216	1.13	0.70	1.82	0.620	1.11	0.69	1.80	0.661

^a^. Adjusted for parity, miscarriage, smoking, obesity

^b^. Norethindrone family includes norethindrone and ethynodiol diacetate; levenorgestrol family includes levonorgestrol, norgestimate, and norgestrel; drospirenone family includes drospirenone

Abbreviations: CI = confidence interval; COC = combined oral contraceptives; MS = multiple sclerosis; CIS = clinically isolated syndrome

## Discussion

In this contemporary cohort, women who developed MS/CIS appear to live a more modern lifestyle than controls. The women who developed MS/CIS were more likely to be current or former smokers and use hormonal contraception. They were less likely to get pregnant, have a baby, or miscarriages/abortions. The use of COCs was independently associated with a slight increased risk of MS/CIS that persisted even after adjusting for known confounders. However, the risk of MS/CIS did not change with increasing duration of COC use, a strong indicator of a non-casual association. While it is possible that the use of some COCs may be contributing at least a small part to the rising incidence of MS in women, unmeasured factors related to the modern woman’s lifestyle, residual confounding or reverse causality are more likely explanations.

Previous studies examining the association between COC and MS have conflicting results. Our findings are consistent with 2 previous prospective cohort studies. A pooled analysis of the Harvard Nurses’ Health Study (HNHS) reported a trend toward an increased risk of MS (relative rate 1.2, 95% CI 0.9–1.5) in ever versus never OC users[2]. Similarly the Royal College of General Practitioners’ Oral Contraception Study reported a trend toward increased risk of MS in former and current users (rate ratio 1.3 95% CI 0.9–2.0; 1.2 95% CI 0.7–2.0, respectively)[3]. These findings did not reach statistical significance in either study which may be due the number of cases included in the analyses (n = 316(2) and n = 114[3], respectively). It should be noted that the authors of these studies had hypothesized a protective effect of OC use and interpreted these findings as evidence of no association between OC use and MS risk. Another prospective cohort[4] study found no association (relative rate = 0.7, 95%CI 0.4–1.1) but was underpowered to detect a difference due to the small number of women who developed MS (n = 63).

Our findings are not consistent with a matched nested case-control study from the General Practice Research Database (GPRD)[5]. This small study (n = 106 MS cases) reported a statistically non-significant decreased risk of MS in women who had used COCs within 3 years of symptom onset compared with those that had not used COCs in this time frame (OR = 0.6, 95%CI 0.4–1.0)[5]. Misclassifying women who used COCs more than 3 years ago as never users may explain these findings.

The most popular hypothesis is that exogenous estrogens like COCs should decrease the risk of MS based on the observation that pregnancy ameliorates disease activity in women with MS and that high dose estrogens ameliorate the animal model of MS (experimental allergic encephalomyelitis; EAE) and have other anti-inflammatory properties. However, this line of reasoning is problematic because contemporary COCs (like those used by the women in our study) contain low doses of estrogen in combination with one of a variety of progestins. The effects of low dose estrogen or progestins, particularly in combination, on EAE or even normal immune responses have not been carefully studied. The relationship between sex hormones and autoimmunity is undoubtedly complex and likely varies based on the doses and combinations of hormones used and the underlying disease.

That COCs could increase the risk of MS/CIS or other autoimmune diseases is biologically plausible. There is some evidence to suggest that low dose estrogen states and COCs could be proinflammatory and enhance autoimmunity. Most autoimmune diseases including MS are more common in women and the onset of disease coincides with the estrous cycle in humans and animals. Women of childbearing age produce a more vigorous immunologic response to vaccinations and infections then men[12]. In vitro studies of proteolipid protein-specific T cell clones from MS patients showed that low doses of estradiol stimulated the proinflammatory cytokines IFNγ and TNFα secretion while high doses stimulated IL-10 secretion[13, 14]. Finally, COC use has been associated with a modest increase in risk of inflammatory bowel disease (IBD)[15, 16]. This is particularly interesting because IBD is the only autoimmune disease with a strong co-morbid association with MS[17].

It is also biologically plausible that the progestins in COC preparations may have different effects on the risk of autoimmunity. Progestins vary in their androgen, progesterone, mineralcorticoid and glucocorticoid receptor affinity and binding properties and could therefore have theoretically diverse effects on MS susceptibility. Levonorgestrol (LNG) is the most androgenic of the progestins contained in modern COCs; norethindrone (NE) has very low androgenic actions and is converted to small amount of ethinyl estradiol; whereas the newest and most unique progestin -drospirenone is a spironolactone-like compound with anti-androgenic effects. No previous studies have examined whether the risk of MS/CIS could vary by progestin content. Interestingly, we found an association with LNG- and NE-containing COCs that was more pronounced among LNG users but no association between drospirenone use and the development of MS/CIS. We speculate that this may be due to the differences in androgenicity of these compounds. However, we had only a small number of cases that used drospirenone so a spurious finding cannot be excluded. This hypothesis should be explored in future studies.

We favor the possibility of unmeasured factors related to a modern woman’s lifestyle (and thereby COC use) as an explanation for our findings. If COC use has a causal relationship to MS and its potential precursor CIS, then it would be expected that the longer the use the higher the risk of MS/CIS. We, like previous studies[2, 5], were unable to demonstrate such a duration of use effect. One potential explanation could be that COCs increase the risk of MS/CIS primarily in the first year after starting analogous to the relationship between COC use and risk of thrombosis[18]. However, we think this type of first-start effect is an unlikely explanation as then the risk of MS/CIS should have *decreased* with longer duration of use.

Aside from COC use, many other aspects of the modern woman’s lifestyle have changed some of which have also been linked to an increased risk of MS or CIS. These include increased prevalence of smoking[19] and obesity[20] and decreased number of offspring[21]. The aspects of a modern woman’s lifestyle that we could measure were indeed more common in the women who went on to develop their first symptoms of MS/CIS and associated with risk in the expected direction.

Another possible explanation is reverse causality- specifically that indications for COC use other than birth control may be more common in women in the prodromal phase of MS. Aside from preventing undesired pregnancies, other indications for COC use include acne and indicators of fertility issues including dysmenorrhea and polycystic ovarian syndrome. A recent Swedish MS case-control study found that both men and women with MS had fewer babies within the 5 but not 10 years prior to diagnosis compared to controls implying that fertility may be impaired during the prodromal phase of MS[22]. Neither we, nor any of the previous studies that also showed a trend towards increased risk of MS with COC use[2, 3] addressed this issue. Future studies should collect information on reason for COC use and fertility histories.

Although we strongly favor a non-causal association, assuming COC use is contributing to the rise in MS incidence we would not recommend a change in COC use policy as over 25,000 women per year would need to be using COCs for 1 additional woman to develop MS/CIS over this period. In addition, several recent studies have reported an association between COC use and a less severe MS disease course[23, 24], milder MS symptoms[25] and older age at onset of first MS symptoms[26]. It should also be noted whether COC use influences the risk of progressive-onset MS (PPMS) is unknown[27] as only one study distinguished between the risk of relapsing and progressive-onset forms and the results were inconclusive as only 9 cases of PPMS were included[5].

Variation in membership duration (and there by COC use information) was an anticipated limitation of this study thus one of the matching criteria for controls was membership duration. We compared the membership duration between ever and never COC users stratified by case/control status to assess for potential differential misclassification. While we did find that those cases and controls classified as never users had a significantly shorter duration of KP membership than ever users this did not vary by case/control status. Thus it does not appear that this bias explains our findings and would be expected to result in an underestimation of the true magnitude of effect. However, we cannot exclude the possibility that COC use prior to KP membership may have been more common among controls than cases. We are currently conducting a study that includes lifetime COC use which will be able to address this issue.

The main limitation of this study is the lack of lifetime COC use and confounder information potentially leading to misclassification of true COC users as never users and multi- as null-parous women. Other limitations include the potential for misclassification of former smokers as non-smokers; restricted range of ethinyl estradiol doses used precluding comparison of true high and low-dose estrogen content; and the frequency of switching between COCs.

Strengths of this study included the biological classification of progestin content; the prospectively collected complete clinical and pharmacy information while members of KPSC; the ability to identify and include CIS cases and matched controls; a cohort that is largely representative of the population from which it is drawn, and the relatively large number of incident CIS/MS cases identified in a short timeframe.

Combined oral contraceptive use was associated with a modestly increased risk of developing MS/CIS and this risk varied by progestin content. However, given the lack of a duration of use effect and the limitations of this and previous studies, this association should be interpreted cautiously as it is most likely due to an unmeasured confounder. Future studies with lifetime COC use, fertility measures and other potential confounders that stratify analyses by progestin content are needed to resolve this question. Our findings do not warrant any change in COC use recommendations.

## Supporting Information

S1 Appendix Baseline demographic and clinical characteristics, MS only cases and matched controls. (DOCX)
