# Supplementary material for: Oral Contraceptives and Multiple Sclerosis/Clinically Isolated Syndrome Susceptibility
Source: PLoS One. 2016 Mar 7;11(3):e0149094. doi: 10.1371/journal.pone.0149094 (PMC4780760; doi:10.1371/journal.pone.0149094)
Supplement: S1 Appendix — (DOCX) [file pone.0149094.s001.docx]

| **Appendix Table 1. Baseline demographic and clinical characteristics, MS only cases and matched controls** | | | | | | | |  |
| --- | --- | --- | --- | --- | --- | --- | --- | --- |
|  |  | **MS Cases (n=239)** | | **MS matched Controls (n=2322)** | | **p-value** |  |  |
|  |  | **n** | **%** | **n** | **%** |  |  |  |
| Age (year), mean (SD) | | 34.2845 | 9.1818 | 34.261 | 9.1779 | 0.970 |  |  |
| Race/Ethnicity, n (%) | |  |  |  |  | 1.000 |  |  |
|  | White | 102 | 42.7 | 992 | 42.7 |  |  |  |
|  | Hispanic | 64 | 26.8 | 629 | 27.1 |  |  |  |
|  | Blacks | 65 | 27.2 | 627 | 27.0 |  |  |  |
|  | Asian/PI | 7 | 2.9 | 65 | 2.8 |  |  |  |
|  | Other | 1 | 0.4 | 9 | 0.4 |  |  |  |
| Body Mass Index, n (%) | | | | |  | 0.907* |  |  |
|  | Under/normal weight | 86 | 36.0 | 827 | 35.6 |  |  |  |
|  | Overweight | 66 | 27.6 | 672 | 28.9 |  |  |  |
|  | Obesity Class I-III | 87 | 36.4 | 823 | 35.4 |  |  |  |
| Smoking, n (%) | |  |  |  |  | 0.040 |  |  |
|  | ever | 78 | 32.6 | 614 | 26.4 |  |  |  |
|  | never | 161 | 67.4 | 1708 | 73.6 |  |  |  |
| Parity, n (%) | | |  |  |  | **0.011** |  |  |
|  | 0 | 198 | 82.8 | 1721 | 74.1 |  |  |  |
|  | 1 | 26 | 10.9 | 404 | 17.4 |  |  |  |
|  | 2+ | 15 | 6.3 | 197 | 8.5 |  |  |  |
| Miscarriage (1+), n (%) | | 18 | 7.5 | 240 | 10.3 | 0.170 |  |  |
|  |  |  |  |  |  |  |  |  |
| Membership duration (months) | |  |  |  |  |  |  |  |
|  | Ever COC users, n | 96 |  | 758 |  | 0.167 |  |  |
|  | median (range) | 120 | (20.0-120.0) | 112 | (12.0-120.0) |  |  |  |
|  | Never COC users, n | 143 |  | 1564 |  | **0.047** |  |  |
|  | median (range) | 93 | (13.0-120.0) | 96.5 | (12.0-120.0) |  |  |  |
| Abbreviations COC=combined oral contraceptives; PI=Pacific Islanders; *under/normal weight vs. overweight or obese | | | | | | | | |
